# Elements of Practical Medicine

**Published:** 1891-06

**Authors:** 


*Elements of Practical Medicine.* By ALFRED H. CARTER, M.D. Sixth Edition. London: H. K. LEWIS. 1891.

This is a new edition, carefully and closely revised, of Dr. Carter's well-known book. The author has received the assistance of Dr. G. F. Crooke and Mr. Malcolm Morris in the preparation of the sections on General Pathology and Diseases of the Skin respectively. The book seems to be well brought up to date, and the fact of its reaching a sixth edition is a sufficient testimonial to its usefulness.

